# Progress in Diseases of the Heart

**Published:** 1901-02-16

**Authors:** 


### PROGRESS IN DISEASES OF THE HEART.

(*Continued from page* 334.)

**Aneurysm.**—F. Jersen[9] quotes two cases in which the "tracheal tug" sign gave misleading evidence. In the first an aneurysm of the aorta was actually pressing upon the trachea and no tug was found. In the second the tug was "easily obtainable" and the post mortem revealed a mediastinal sarcoma and no aneurysm.

De Havilland Hall[10] lays stress upon the diastolic shock in cases of thoracic aneurysm, but doubts whether or no tracheal tugging is a sign to be relied upon. The discussion thus introduced, induced remarks from Sir William Broadbent, who spoke to the value of potassium iodide, which in his opinion made Tuffnell's treatment unnecessary. Dr. Maguire attested to the value of Laucereaux's gelatine treatment. Four cases he had treated with good results. He used injections into the gluteal muscles under local anæsthesia, of 100 c.c. of absolutely sterile 2 per cent. gelatin solution in 7 per 1,000 NaCl solution. He gave four consecutive injections in four days, waited for a fortnight and then repeated that treatment. He held that a great deal of the danger was due to sepsis and the risk of tetanus. With aseptic precautions the risks should be much lessened.

Dr. Kingston Fowler[11] considered that tracheal tugging when present was diagnostic of aneurysm. In one case in which Lancereaux's treatment had been tried embolism of the internal carotid ensued, whether due to the treatment he could not say. In two others it had no result.

Dr. Gilbert Smith[11] stated that in a case where tracheal tugging had occurred clinically the necropsy revealed a dilated pulmonary artery and a shrunken lung, but no aneurysm.

Dr. Seymour Taylor[11] concludes that tracheal tugging is of no diagnostic value.

Walsham[12] describes the cases of two patients, and reports a third, in whom aneurysm was discovered by means of the Roentgen rays, the diagnosis being quite impossible by other clinical methods.

Stengel[13] gives the notes of a case of aneurysm of aorta which was fatal owing to rupture taking place, and compression of the superior vena cava being caused thereby. Enormous œdema and ecchymoses were the result, and finally death in convulsion.

**Drugs.**—Prof. Zenetz[14] states that caffeine used in large doses is a decidedly dangerous remedy. Death supervenes suddenly with an arrest of the heart in systole, which is the contrary to what is observed in animals, where the heart is arrested in disastole. He quotes a case where a young woman poisoned herself with repeated small doses of caffeine—she first fell insensible, but was restored by artificial respiration and bleeding; later she continued to take more caffeine and fell suddenly dead. At the autopsy the heart was so contracted and hard as to be cut with difficulty. Amount taken was ten powders of 3 decigr. each.

It is especially dangerous, he says, in the fibrinous pneumonia of the aged. He gives a case of sudden death arising from its use. In this case also the heart had been arrested in systole. The dose used had been 2 decigr. taken for two days.

A third case, one of chronic nephritis, took a dose of 2 grammes of sodium benzoate of caffeine in 180 grammes of water. After each dose she felt better, but after taking it five or six times she died suddenly, with heart arrested in systole.

Caffeine is cumulative in some patients; and after giving the drug (2 or 3 decigrammes a day) for 15 or 16 days, he replaced it with bicarbonate of soda, and caffeine was excreted in the urine from 10 to 15 days longer.

Zeultz maintains that "continued treatment with caffeine is only permissible so long as it is not recoverable from the urine. He also says that while it is slowly excreted it is easily stored up, and if taken continuously it can suddenly produce toxic symptoms. This is especially so in cases of renal disease, and thus caffeine should not be counted a "true diuretic," because of its danger in cases where diuretics are needed most. It is also contra-indicated in arterial sclerosis. He states that what he says is applicable to the salts of caffeine as to the drug itself.

J. A. MacLaren[15] reports his conclusions after studying the action of digitalis, strophanthus, and diuretin as diuretics upon himself. He states that digitalis if pushed till toxic conditions manifest themselves, is a direct diuretic, that strophanthus acts as a diuretic solely by its tonic effect upon the heart, and that diuretin has possibly a slight diuretic action in health, which is not produced by any effect on the heart or circulation, but by its stimulating effect upon the secretory epithelium of the kidney. The latter, however, has a most nauseous taste, is expensive, and causes in nearly every case unpleasant gastro-intestinal symptoms.

He points out that rest in bed is followed by diuresis in 60 per cent. of cases, usually on the third day, without the use of drugs, and this may lead to a fallacious idea of the diuretic property of drugs. He explains this from the increase of cardiac power from the recumbent position and the regularity of hospital diet.

With the use of digitalis clinically in cardiac dropsy, he found that the excretion of solids and fluids increased, the urea still increasing after the withdrawal of digitalis. Diuresis set in on the third day generally. (With Nativelle's digitalin on the second day.)

Strophanthus was not so efficient and caused gastro-intestinal trouble.

With diuretin diuresis set in within 24 hours after the administration of the drug in half the cases. Toxic symptoms were produced easily.

With regard to urea digitalis produces a large increase in the daily elimination in 23 out of 25 cases, and in his opinion acts directly upon the renal tubular epithelium. He found that a temperature of over 100° Fahr. hindered the diuretic action of those drugs. He states that in the anasarca of Bright's disease, diuretin "does undoubtedly act more favourably than either digitalis or strophanthus.'

[8] Med. Rec., Dec. 1. [10] Lancet, Dec. 1. [11] Brit. Med. Jour., Dec. 15. [12] Lancet, Nov. 3. [13] Amer. Jour. Med. Sci., November. [14] Les Nouveaux Remédies, Oct. 8. [15] Med. Rec., September.

